# Atypical Endoscopic Appearance of a Rare Cause of Gastrointestinal Bleeding: Superior Mesenteric Vein Occlusion Causing Colon Varices

**DOI:** 10.14309/crj.0000000000001680

**Published:** 2025-04-30

**Authors:** Misha Gautam, Vinay Jahagirdar, Kimia Zoraghchi, Thomas Cunningham, Mohamed Ahmed, Ben Aziz, Nathan Saucier, John P. Campbell

**Affiliations:** 1 Department of Internal Medicine, University of Missouri-Kansas City School of Medicine, Kansas City, MO; 2 University of Missouri-Kansas City School of Medicine, Kansas City, MO; 3 Department of Gastroenterology, Saint Luke's Hospital of Kansas City, Kansas City, MO; 4 Department of Interventional Radiology, Saint Luke's Hospital of Kansas City, Kansas City, MO

**Keywords:** colonic varices, superior mesenteric vein occlusion, non-cirrhotic portal hypertension, coil embolization, gastrointestinal bleeding

## Abstract

Colonic varices (CV) are an uncommon cause of gastrointestinal bleeding, with an incidence of only 0.07%. We present a case of a 34-year-old woman with chronic superior mesenteric vein occlusion after pancreatoduodenectomy, leading to diffuse colonic varices with an atypical appearance needing further evaluation. Subsequent angiography confirmed CV, which were treated with catheter-based superior mesenteric vein stenting and variceal coiling. The patient's bleeding was controlled, with plans for continued surveillance. This case emphasizes the diagnostic challenges, treatment complexities, and the need for individualized management strategies for CV, highlighting the urgent need for established guidelines.

## INTRODUCTION

Colonic varices (CV) are a rare cause of lower gastrointestinal bleed (GI), especially so in patients without liver cirrhosis. Although established guidelines are lacking, the management is often treating the underlying cause of increased collateral mesenteric blood flow. We present a unique case of high-risk CVs secondary to superior mesenteric vein (SMV) occlusion, which were safely treated with catheter-based stenting and variceal coiling. Providers should be aware of this life-threatening condition and the various approaches that can be used for treatment.

## CASE REPORT

**Figure 1.**
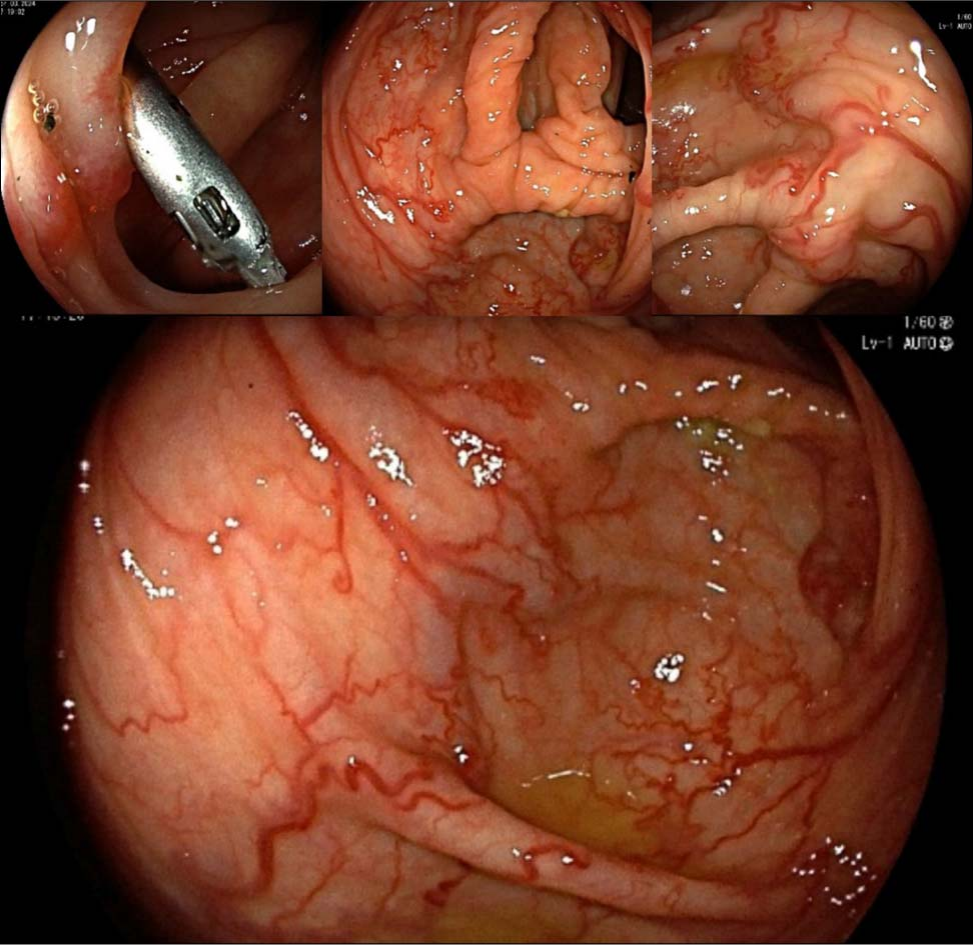
Colonoscopy showing dilated tortuous nonbleeding submucosal vessels, and the clip deployed for marking for future reference.

**Figure 2.**
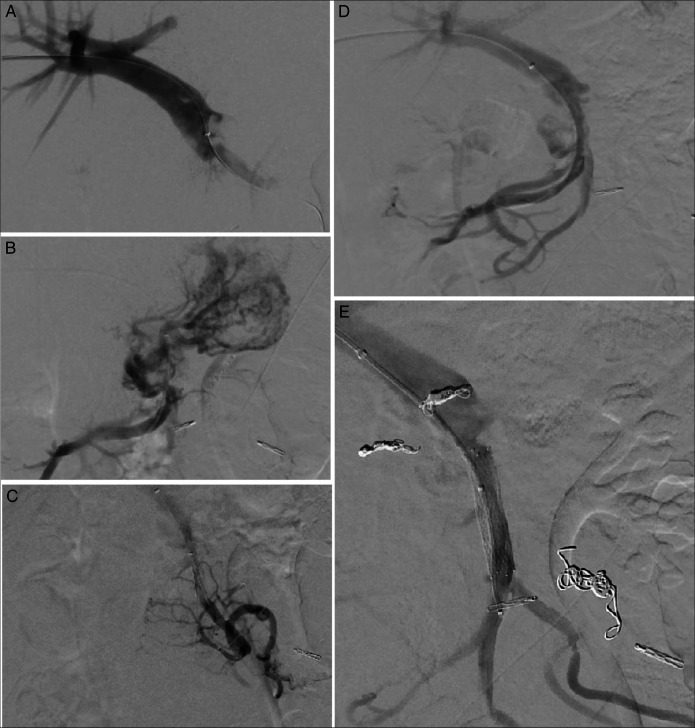
(A) Portal venogram demonstrating severe narrowing at the superior mesenteric vein/portal confluence. (B) and (C) Increased pressure in the mesenteric veins and causing gastric and colonic varices, respectively. (D) Angioplasty and bare metal stent placement resulted in significantly improved flow. (E) Coils are placed in multiple varices with significantly decreased flow.

A 34-year-old woman with a medical history of Tetralogy of Fallot status post repair and pulmonary conduit, mild aortic regurgitation, recurrent GI bleeding from duodenal arteriovenous malformations (AVMs), intestinal malrotation, history of Whipple's procedure (at age of 9 years) was admitted for symptomatic anemia (hemoglobin 5.8 g/dL) requiring blood transfusion. She denied abnormal menstrual bleeding or overt GI bleeding. Her iron studies, vitamin B12, and folate were within normal limits. She was taking an iron supplement at home, causing chronically dark stools, obscuring evaluation for melena. Given her history of bleeding intestinal AVMs, a proton pump inhibitor was started, and combined esophagogastroduodenoscopy and colonoscopy were planned for the next day. Esophagogastroduodenoscopy showed localized congestion and erythema of the fundic mucosa compatible with gastritis. The biopsies taken for *Helicobacter pylori* testing were negative. No bleeding source was identified. The colonoscopy revealed nonbleeding engorged vessels at 60 cm from the anal verge, concerning for massive AVMs on this initial evaluation. These involved 4 to 5 cm of circumferential colonic mucosa in the hepatic flexure territory (Figure 1). Too diffuse to be endoscopically treated, a decision was made to obtain computed tomography angiography of the abdomen and pelvis, which demonstrated short segment occlusion of the proximal SMV and prominent vessels within the wall of the ascending colon extending over at least 5 cm in length located between 2 endoscopically placed clips. It also revealed multiple small arterial phases enhancing lesions throughout the liver largest measuring up to 1.6 cm (possibly reflecting multiple small hepatic telangiectasias, hepatic perfusion abnormalities/vascular shunts, or benign lesions such as FNH or adenomas). Her post-transfusion hemoglobin remained stable at 10.4 with no overt GI bleeding. She was thus discharged with plans for small bowel video capsule endoscopy and close follow-up with interventional radiology for intervention. Elective balloon angioplasty and bare metal stenting of the severe narrowing at the proximal SMV were performed. The multiple mesenteric and CVs were coil embolized with stasis of flow achieved (Figure 2). She remained on anticoagulation and surveillance with abdominal and pelvic computed tomography with portal venous phase in 4–6 weeks. However, 5 months later, the patient was readmitted with melena and a hemoglobin level of 3.6. She was found to have bleeding jejunal ulcers and small nonbleeding ascending colon angioectasias, which were treated with hemostatic clips and argon plasma coagulation, respectively.

## DISCUSSION

The incidence of GI bleeding because of CV is merely 0.07%.^1^ Based on the cause of the portomesenteric hypertension leading to the varices, the site may vary from inferior mesenteric vein, SMV, or mixed supply distribution. The most common etiology is the portal hypertension secondary to liver cirrhosis; however, other etiologies such as venous thrombosis/occlusion, congestive heart failure, pancreatitis, and postpancreatoduodenectomy have been seen.^2,3^ Our patient presented with multiple gastrointestinal and cardiac anomalies that significantly impacted her hemodynamics and vascular flow patterns. Her underlying congenital heart disease, specifically Tetralogy of Fallot, predisposed her to thrombotic events. In addition, her intestinal malrotation and history of pancreaticoduodenectomy contributed to altered vascular anatomy and elevated mesenteric venous pressure, encouraging the formation of collateral vessels. These chronic vascular stresses likely facilitated the development of AVMs and SMV thrombosis. Furthermore, the Whipple procedure is known to induce sinistral (left-sided) portal hypertension, further promoting collateral venous pathways that commonly present as gastric, esophageal, or CVs.

**Figure 3.**
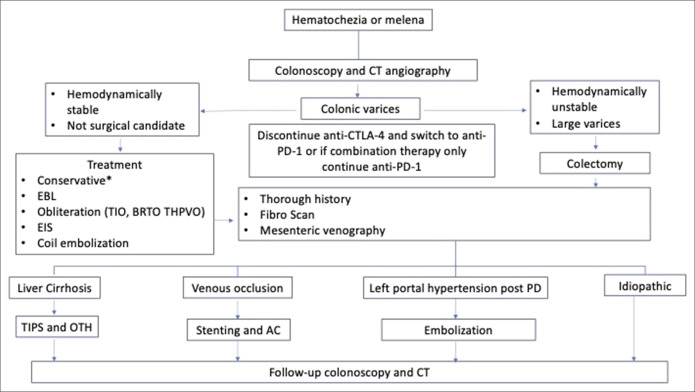
Diagnostic and therapeutic options for symptomatic colonic varices. *Conservative medical management with beta-blockers and somatostatin (octreotide). BRTO, balloon-occluded retrograde transvenous obliteration; CT, computed tomography; EBL, endoscopic band ligation; EIS, endoscopic injectable sclerotherapy; OTH, orthotic liver transplant; THPVO, transhepatic portal venous approach to obliteration; TIO, transileocolonic vein obliteration; TIPS, transhepatic intravenous portosystemic shunt.

CV may be an incidental finding with routine colonoscopy, or patients are symptomatic with hematochezia/anemia. Although colonoscopy is often the first diagnostic modality used, the detection may be obscured with inadequate prep, active bleeding, or missed if varices are flattened with insufflation or worse mistaken for a polyp or tumor. In our case, the CV were not engorged, but diffuse, and endoscopically assessed to be an extensive AVM. The standard for diagnosis and eliciting the cause for CV is angiography.

The treatment of CVs is not standard owing to the low incidence, variable etiologies, and anatomic patterns. Based on individual cases, different interventions can be used based on the acuity of presentation and patient-physician discretion. These interventions generally include a variety–conservative approach (beta-blockers), transhepatic intrajugular portosystemic shunt, balloon occluded transvenous obliteration, endoscopic variceal ligation, somatostatin infusion, argon plasma coagulation, sclerotherapy, coil embolization, transvenous obliteration, colectomy, and decreasing portomesenteric hypertension with stenting.^4–10^ A single intervention or a combination may be used to treat the varices and reduce chances of reoccurrence (Figure 3). Close follow-ups with imaging or endoscopy are crucial to check for colon necrosis and reoccurrence.

CV are a rarely encountered entity, however, when present can induce life-threatening hemorrhage. There is an urgent need for better risk stratification in patients with CV. In addition, screening and management guidelines need to be established.

## DISCLOSURES

Author contributions: M. Gautam, V. Jahagirdar, K. Zoraghchi, T. Cunningham, J. Campbell: conception or design of the work and acquisition and interpretation of data for the work. M. Gautam: drafting the work. M. Ahmed, B. Aziz, N. Saucier, J. Campbell: reviewing it critically for important intellectual content and final approval of the version to be published, ensuring that questions related to the accuracy are appropriately investigated. J. Campbell is the article guarantor.

Financial disclosure: None to report.

Informed consent was obtained for this case report.
